# Lower SLC7A2 expression is associated with enhanced multidrug resistance, less immune infiltrates and worse prognosis of NSCLC

**DOI:** 10.1186/s12964-022-01023-x

**Published:** 2023-01-13

**Authors:** Shanshan Jiang, Junrong Zou, Jianyu Dong, Huimian Shi, Jie Chen, Yan Li, Xianglong Duan, Wensheng Li

**Affiliations:** 1 Institute of Hematological Research, Shaanxi Provincial People’s Hospital, 256 West Youyi Road, Xi’an, 71000 Shaanxi China; 2 The First Affiliated Hospital of Gan’nan Medical University, Ganzhou, China; 3 Breast Center, Department of General Surgery, Nanfang Hospital, Southern Medical University, Guangzhou, China; 4 Yiling Pharmaceutical Co., Ltd, Shijiazhuang, China; 5 Shaanxi Provincial Key Laboratory of Infection and Immune Diseases, Shaanxi Provincial People’s Hospital, Xi’an, China; 6 Second Department of General Surgery, Shaanxi Provincial People’s Hospital, 256 West Youyi Road, Xi’an, 71000 Shaanxi China; 7 Institute of Medical Research, Northwestern Polytechnical University, Xi’an, China; 8 Second Department of General Surgery, Third Affiliated Hospital of Xi’an Jiaotong University, Xi’an, China; 9 Department of Pathology, Shaanxi Provincial People’s Hospital, 256 West Youyi Road, Xi’an, 71000 Shaanxi China

**Keywords:** SLC7A2, Multiple drug resistance, NSCLC, Immune infiltration, AMPK

## Abstract

**Background:**

Solute carrier family 7 member 2 (SLC7A2), a cationic amino acid transporter, is lowly expressed in ovarian and hepatocellular cancers, which is associated with their worse prognosis. However, its roles in the prognosis, drug resistance and immune infiltration in non-small-cell lung cancer (NSCLC) are unclear.

**Methods:**

We chose SLC7A2 from RNA-Seq of paclitaxel/cisplatin-resistant A549 cells, then bioinformatics, cell lines construction, RT-qPCR, and CCK8 were performed to investigate SLC7A2 role.

**Result:**

We analyzed the 223 differentially expressed genes (DEGs) from RNA-Seq of paclitaxel/cisplatin-resistant A549 cells and found that SLC7A2 expression was down-regulated in NSCLC. Lower SLC7A2 expression was associated with worse recurrence-free survival (RFS) in NSCLC. SLC7A2 silencing enhanced the proliferation of NSCLC cells and their insensitivity to paclitaxel, cisplatin, and gemcitabine in vitro. Activation of AMPK has up-regulated SLC7A2 expression and enhanced the sensitivity of NSCLC cells to anti-tumor drugs, which could be attributed to E2F1’s regulation. In addition, the levels of SLC7A2 expression were correlated to the numbers of infiltrated neutrophils, macrophages, dendritic cells and their marker genes, like CD86, HLA-DPA1 and ITGAM.

**Conclusions:**

SLC7A2 may act as a tumor suppressor to modulate drug sensitivity, immune infiltration and survival in NSCLC.

**Video abstract**

**Supplementary Information:**

The online version contains supplementary material available at 10.1186/s12964-022-01023-x.

## Introduction

Lung cancer still has the highest mortality rate worldwide [1]. Non-small-cell lung cancer (NSCLC, 85% of total lung cancers) is the main type of lung cancer [2]. Although therapeutic advancements, such as targeted therapy and immunotherapy, have recently increased the survival rates of NSCLC [1], many patients with NSCLC due to the lack of suitable mutants or insensitivity to immunotherapies still depend on chemotherapy with paclitaxel, cisplatin, gemcitabine [3, 4]. Unfortunately, the development of chemoresistance to these drugs remains a barrier to affect their efficacy and prognosis of NSCLC. Hence, it is of importance to understand the molecular mechanisms underlying the development of chemoresistance to benefit NSCLC patients.

The solute carrier family 7 member 2 (SLC7A2, also known as CAT2) is a member of the solute carrier superfamily. Currently, SLC7A2 can functionally transport cationic amino acids, like L-arginine, into the cytosol and regulate inflammation [5]. Recent studies have shown that lower SLC7A2 expression is associated with worse prognosis of ovarian cancer and hepatocellular carcinoma [6, 7], and SLC7A2 deficiency in inflammatory bowel tissues increases the risk of inflammation-associated colon tumorigenesis [8]. These suggest that SLC7A2 may act as a tumor suppressor during the development of malignant tumors. A recent bioinformatic analysis indicates that SLC7A2 is associated with the development of NSCLC [9] and up-regulated SLC7A2 expression is related to radioresistance of NSCLC cells [10]. Hence, SLC7A2 expression profiles and functions in the proliferation, drug-resistance and immune infiltration in NSCLC are still unclear.

In this study, we analyzed transcriptomes of wild-type and drug-resistant A549 cells by RNA-seq and explored SLC7A2 expression profiles and associated recurrence-free survival (RFS) of NSCLC patients in available databases and patient’s tissues. Subsequently, we tested the potential functions and mechanism of SLC7A2 in the proliferation and drug sensitivity of NSCLC cells and how activation of AMPK modulated SLC7A2 expression. Finally, we explored the potential association between the levels of SLC7A2 gene transcripts and immune infiltration in NSCLC.

## Materials and methods

### Materials

Special reagents used in this study included paclitaxel, cisplatin, fluorouracil, gemcitabine, methotrexate vinblastine and Cell Counting Kit-8 (CCK-8) (Good Laboratory Practice Bioscience, Montclair, CA, USA), oxaliplatin and saracatinib (Selleck, Shanghai, China); anti-SLC7A2, anti-β-actin (ABclonal Technology, Wuhan, Hubei, China), anti-phosphorylated AMPK, anti-AMPK (Cell Signaling Technology, Boston, MA, USA); the shRNA-SLC7A2 and control plasmids (Genechem, Shanghai, China); the RNeasy Mini Kit and QIAGEN Nova SYBR Green PCR Kit (Qiagen, Germantown, MD, USA), and FastKing RT Kit (with gDNase) (Tiangen Biotech, Beijing, China).

### Methods

#### RNA sequencing assay

The RNA sequencing assay was performed by Gene Denovo Biotechnology (Guangzhou, China). Total mRNAs were extracted from each cell line using Trizol reagent kit (Invitrogen, Carlsbad, CA, USA), according to the manufacturer’s protocol (Invitrogen, Carlsbad, CA, USA). The RNA quality was evaluated by Agilent 2100 Bioanalyzer (Agilent Technologies, Palo Alto, CA, USA) and RNase free agarose gel electrophoresis. The total mRNAs were further enriched by Oligo(dT) beads (Epicentre, Madison, WI, USA) and fragmented into short fragments (about 200 nucleotides) using fragmentation buffer. Subsequently, the fragmented mRNAs were reverse-transcribed into cDNA using random primers, DNA polymerase I, RNase H, dNTP and buffer. After purification, repairing, ligating the cDNA with the Illumina sequencing adapters, agarose gel electrophoresis and PCR assay, the products were sequenced using Illumina Novaseq 6000 (Gene Denovo Biotechnology, Guangzhou, China).

#### Oncomine database analysis

The relative levels of *SLC7A2* mRNA transcripts in various types of cancer and normal tissues were analyzed by Oncomine database (https://www.oncomine.org) [11] and the differentially expressed genes (DEGs) were identified using thresholds of *p* ≤ 0.001 and fold change = 1.5.

#### Gene ontology (GO) and Kyoto encyclopedia of genes and genomes (KEGG) analysis

The potential functions of DEGs were analyzed by GO functional enrichment analyses using Omicshare Online tools (https://www.omicsmart.com/) and three ontologies of molecular function (MF), cellular component (CC) and biological process (BP) for GO analyses. The potential enriched pathways of DEGs were analyzed by KEGG.

#### PrognoScan database analysis

The correlation between SLC7A2 expression and the RFS of patients with different types of cancers was analyzed using PrognoScan database (http://dna00.bio.kyutech.ac.jp/PrognoScan/).

#### Tumor immune estimation resource (TIMER) database analysis

The immune infiltrates in various types of cancers were analyzed using TIMER Database (https://cistrome.shinyapps.io/timer/) [12]. The correlation between SLC7A2 expression and immune infiltrates was analyzed and immune infiltrates included B cells, CD8+ T cells, CD4+ T cells, macrophages, neutrophils and dendritic cells and their markers [13, 14]. The relationship between SLC7A2 expression and macrophage polarization was also analyzed using a threshold of COX *p* ≤ 0.05.

#### Stable cancer cell lines

To generate lentivirus, 293 T cells were transfected with pGV654-shRNA-SLC7A2 or Control plasmid, together with lentivirus packaging plasmids using lipofectamine 2000 for 48 h. Their supernatants were collected and filtered through 0.22 mm Millipore filters, followed by ultracentrifuge. NSCLC cells were transduced with each type of lentivirus at MOI of 5 in the presence of polybrene and treated with 500 µg/ml G418 to establish stable SLC7A2-silenced cell lines.

#### Cisplatin resistant cancer cell lines

3 × 10^6^ A549 cells were seeded into one 10 cm dish. Next day, cells were then treated with drug of specified concentration for 15 days. If the cells grew well, we then added the drug with further elevated concentration; if over half of the cells were killed, we should stop drug usage until the number of cells regain 3 × 10^6^. Following this order, we added cisplatin with concentrations of 10, 20, 40, 50, 60, 70, 80, 90 µM. Cisplatin powder was dissolved with dimethylformamide, resulting in 10 mM cisplatin stock solution. And the concentration of paclitaxel is 10, 20, 30, 40, 60, 70, 90, 100 nM. The paclitaxel powder was dissolved with dimethylsulfoxide and the stock solution concentration is 10 µM.

#### CCK-8 assays

Drug-sensitive and resistant A549 cells (1500 cells/well) were cultured in triplicate in 96-well plates overnight and treated with different concentrations of individual drugs at a specific concentration for varying time periods. During the last one-hour culture, each well was added with 10 µl of CCK-8. The absorbance at 450 nm in individual wells was measured using a microplate reader.

#### Clone formation assay

Drug-sensitive and resistant A549 cells (500 cells/well) were cultured in 6-well plates, and treated with, or without, metformin (10 mM) for 15 days. The formed clones were stained with Wright and Giemsa solution and photoimaged.

#### Western blot

The cells were harvested and lysed in RIPA lysis buffer. After determination of protein concentrations, the cell lysate samples (20 µg/lane) were separated by 10% SDS-gel electrophoresis, and transferred onto PVDF membranes. The membranes were blocked with 5% no-fat dry milk for 1 h and incubated with primary antibodies overnight at 4 °C. Subsequently, the bound antibodies were detected with second antibodies for 1 h at room temperature and visualized using FluorChem HD2 (Alpha Innotech, San Francisco, CA, USA).

#### Reverse transcription quantitative polymerase chain reaction (RT-qPCR)

Total mRNA was extracted from each type of cells and reverse-transcribed into cDNA using RNeasy Mini Kit, according to the manufacturer’s instructions. The relative levels of targeted gene mRNA transcripts to the control GAPDH were quantified by RT-qPCR using SYBR Green Master, specific primers, and cDNA template. The specific primers were forward 5′–CCCGGGATGGCTTACTGTTT–3′ and reverse 5′–CAAAGCAGAAATGACCCCTGC–3′ for SLC7A2 (a product of 95 bp); forward 5′–CAGGGCTGCTTTTAACTCTGGT–3′ and reverse 5′–GATTTTGGAGGGATCTCGCT–3′ for GAPDH (a product of 199 bp), forward 5′–AGTACCTGAGCTCGCCAGT–3′ and reverse 5′–AAGCCCTTGCTGTAGTGGTG–3′ for interleukin 1 beta (IL-1β, a product of 174 bp), forward 5′–TCCCCTGAGGCATTTAGGCA–3′ and reverse 5′–GAAAAGGCTCCCAGGGCTAA–3′ for toll like receptor 4 (TLR4, a product of 177 bp), forward 5′–ACCTGGGAAATTCAAGGCGT–3′ and reverse 5′–CCGAAGTGCAGATTCCCTCC–3′ for complement C5 (C5, a product of 220 bp), forward 5′–CAGACCACGCAAGGAGTTCA–3′ and reverse 5′–CTTCCACCTTGGAGCACTGT–3′ for C-X-C motif chemokine ligand 5 (CXCL5, a product of 82 bp), forward 5′–GAGGCTCTGAAGGTCCCCA–3′ and reverse 5′–GGGCCAGCAGCACTAGC–3′ for collagen type I alpha 1 chain (COL1A1, a product of 70 bp), The amplification reactions were performed in duplicate and subjected to 40 cycles of 95 °C for 2 min, 95 °C for 5 s, 60 °C for 10 s,. The results were calculated by 2^−ΔΔCT^.

#### Co-inmunoprecipitation (Co-IP)

The protein of 2 × 10^7^ H460 cells were extracted by 2 ml cell lysis buffer for Western and IP (Beyotime, Beijing, China), and then kept 100 µl as pre-IP, 20 µl primary SLC7A2 antibody was incubated with 10 µl protein A/G beads at 4 °C for 4 h. Next, we added the rest protein into antibody- A/G beads overnight at 4 °C. The next day, the protein- antibody-A/G beads were centrifuged at 12,000 g for 5 min. The supernatant was post-IP and the deposit was IP. At last, the E2F1 and SLC7A2 protein were detected by Western blot.

#### Immunohistochemistry (IHC)

The IHC assay was performed as described before [15]: briefly, the NSCLC tissues were retrieved by citric acid buffer and incubated with SLC7A2 primary antibody at the dilution of 1:100 (A14574, Abclonal, MA, USA) overnight at 4 °C, then incubated with second antibody at 37 °C for 1 h (PV-6000, ZSGB-BIO, Beijing, China). After color development and counterstain with 3,3′‑diaminobenzidine and hematoxylin, the staining intensity and percentage of positive staining were evaluated by semi-quantitative analysis using light microscope according to the immunoreactive score (IRS).

## Statistical analysis

All the data are expressed as mean ± SEM. The difference between groups was analyzed by Student’s *t*-test, one-way ANOVA using the GraphPad Prism software, Chi-square using the SPSS statistics. A *p*-value of < 0.05 was considered significant difference.

## Results

### Establishment of drug-resistant A549 cells

**Fig. 1:**
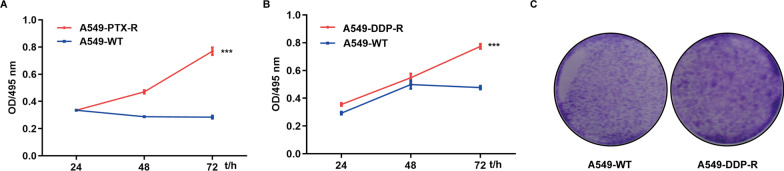
Establishment of paclitaxel- and cisplatin-resistant A549 cells. A549 cells were gradually treated with increased concentrations of paclitaxel or cisplatin for two weeks per dose. When they are resistant the highest dose tested and they were tested for proliferation and clone formation. **A** and **B** CCK-8 assay determined the dynamic proliferation of the indicated cells in the presence of the drug. **C** Representative images of clone formation of the indicated cells (magnification × 100). ***p < 0.001

Drug resistance is a common reason for chemotherapy failure. To explore the potential mechanisms underlying drug resistance, we established paclitaxel-resistant A549-PTX-R as a previous report [16] and cisplatin-resistant A549-DDP-R cell lines. Clearly, treatment with 100 nM paclitaxel or 90 µM cisplatin inhibited the proliferation of control A549 cells while A549-PTX-R and A549-DDP-R cell lines displayed their proliferation with time, a hallmark of drug resistance (Fig. 1A, B). Moreover, the clone formation assay revealed that A549-DDP-R cells grew faster than A549-WT cells (Fig. 1C). Hence, A549-PTX-R and A549-DDP-R cells were drug resistant cells.

### RNA-seq analysis of paclitaxel- and cisplatin-resistant A549 cells

**Fig. 2:**
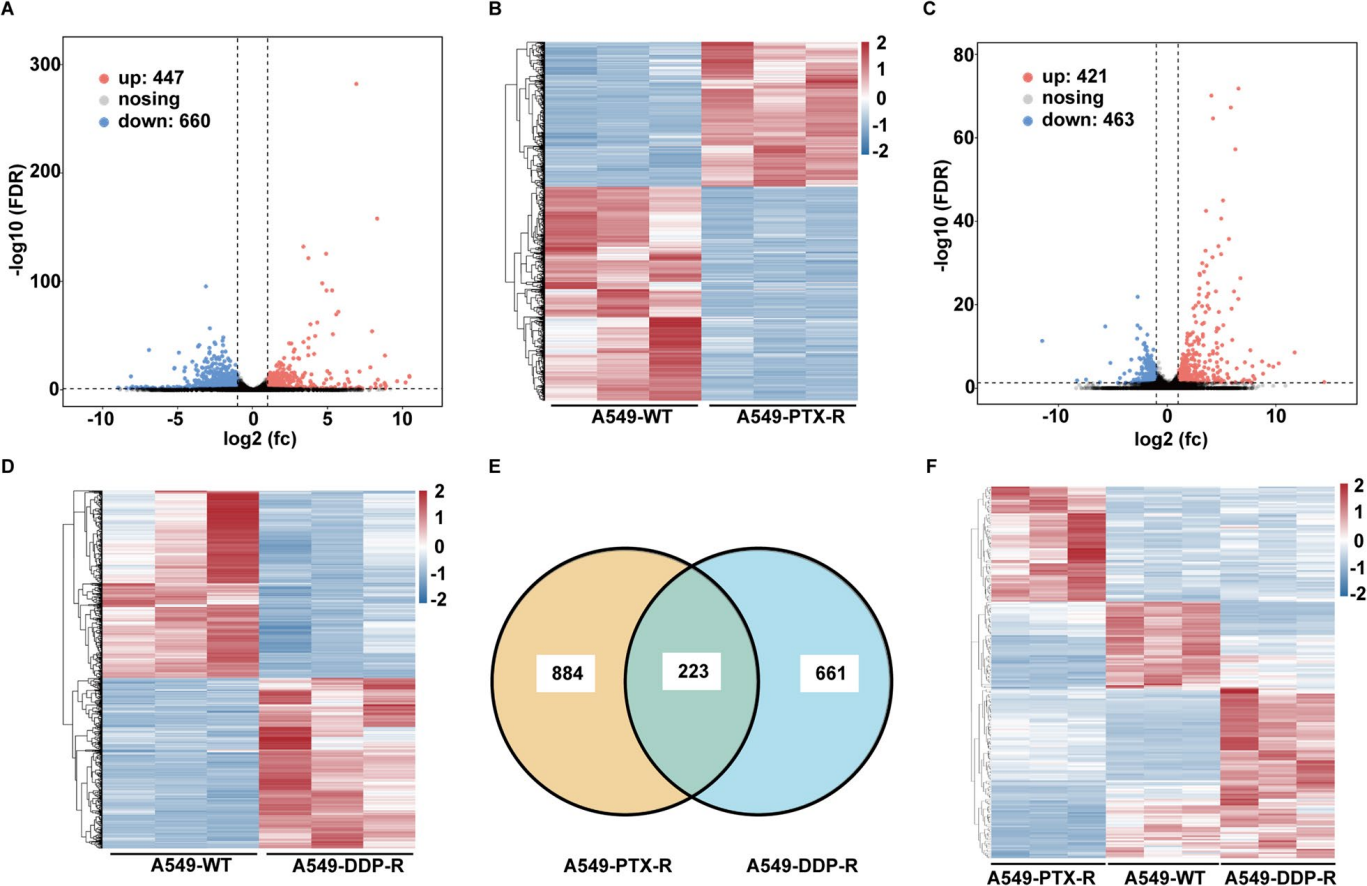
Analysis of 223 DEGs in the paclitaxel- and cisplatin-resistant A549 cells. **A** Volcano analysis of the DEGs between A549-PTX-R and wild-type A549 cells. **B** The heatmap analyses of the DEGs between A549-PTX-R and wild-type A549 cells. **C** Volcano analysis of the DEGs between A549-CIS-R and wild-type A549 cells. **D** The heatmap analyses of the DEGs between A549-CIS-R and wild-type A549 cells. **E** There are 223 DEGs in both paclitaxel- and cisplatin-resistant A549 cells. **F** The heatmap analyses of the 223 DEGs in both paclitaxel- and cisplatin-resistant A549 cells

To understand the potential mechanisms underlying the development of drug resistance, we analyzed the difference in transcriptomes between wild-type and drug-resistant A549 cells by RNA-seq. There were 43,423,127 ± 4,756,524 clean raw reads from cisplatin-resistant cells, 66,035,803 ± 2,190,052 raw reads from paclitaxel-resistant cells and 51,206,167 ± 4,826,313 from A549 wildtype cells. There were 1107 DEGs between paclitaxel-resistant and WT cells. Among them, 447 genes were upregulated and 660 downregulated (Fig. 2A, B). There were 884 DEGs between cisplatin-resistant and WT cells. Among them, 421 genes were upregulated and 463 down-regulated (Fig. 2C, D). There were 223 DEGs between the paclitaxel/cisplatin-resistant and WT cells (Fig. 2E, F).

**Fig. 3:**
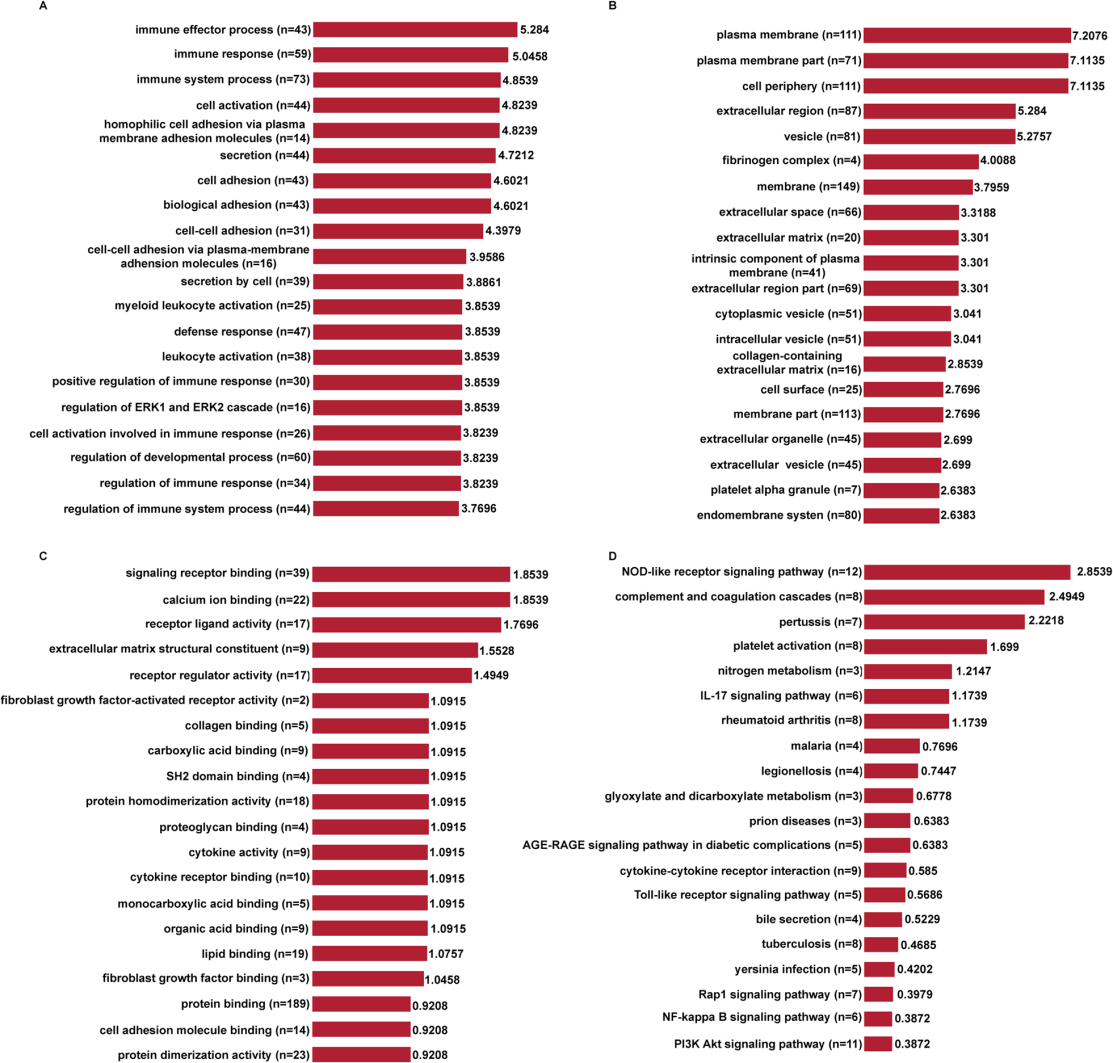
The functional enrichment analysis of the 223 DEGs. **A** The top 20 biological process terms in the GO analysis. **B** The top 20 cellular component terms in the GO analysis. **C** The top 20 molecular function terms in the GO analysis. **D** The top 20 pathways in the KEGG analysis

To explore the potential functions of these DEGs, GO analyses indicated that these DEGs were enriched in immune effector process, immune response and immune system process of the BP (Fig. 3A); plasma membrane, plasma membrane part and cell periphery of the CC (Fig. 3B); and signaling receptor binding, calcium ion binding and receptor ligand activity of the MF (Fig. 3C). The KEGG analysis revealed that the NOD-like receptor signaling pathway was the most enriched signaling pathway and coagulation system pathways were also related to paclitaxel and cisplatin resistance (Fig. 3D).

### The validation of KEGG pathways related genes in paclitaxel and cisplatin resistant cell lines

**Fig. 4:**
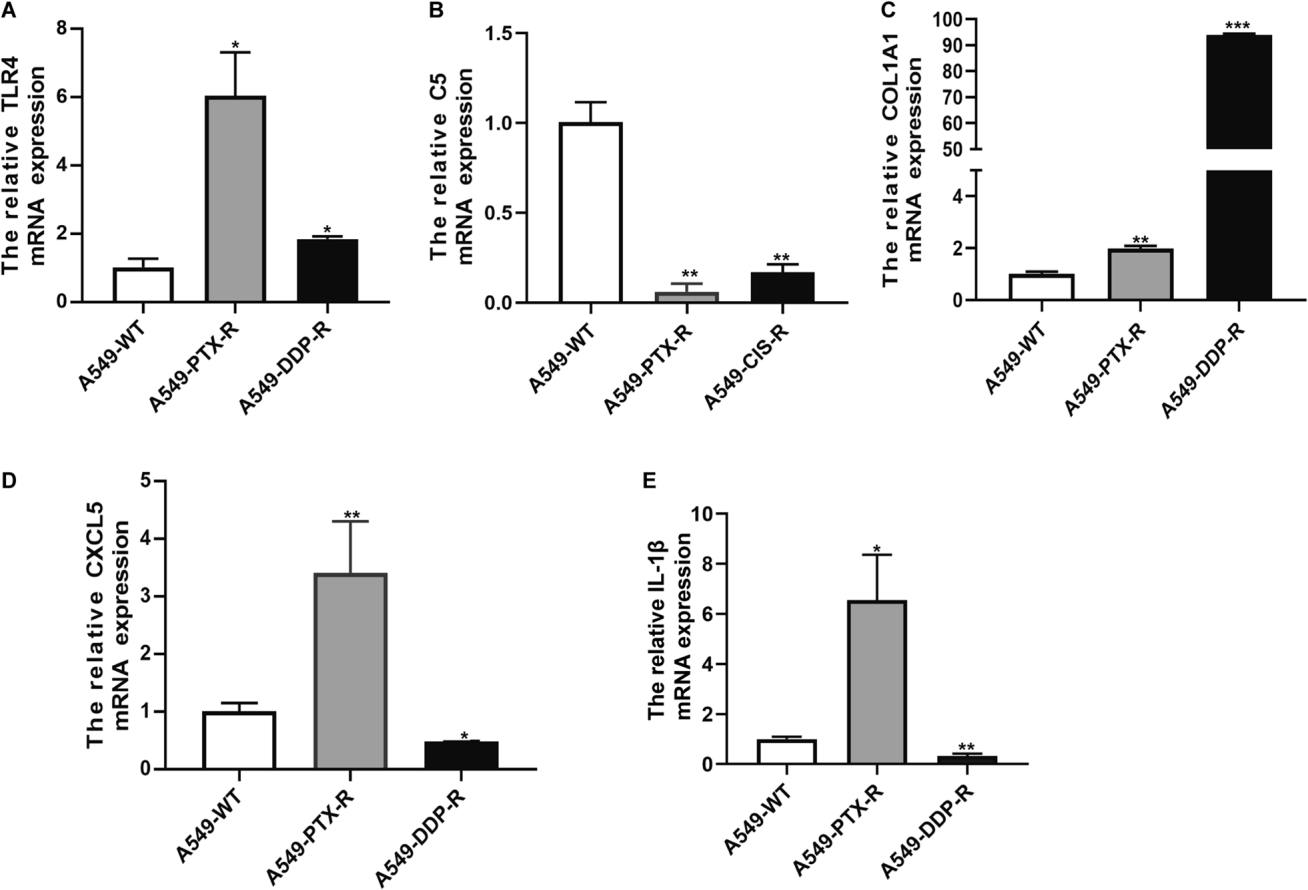
The validation of KEGG pathways related genes in paclitaxel and cisplatin resistant cell lines. The relative TLR4 (**A**), C5 (**B**), COL1A1 (**C**), CXCL5 (**D**) and IL-1β (**E**) mRNA expression in paclitaxel and cisplatin resistant A549 cell lines. *p < 0.05; **p < 0.01; ***p < 0.001

We have analyzed the top 20 pathways in KEGG pathways (Fig. 3D) and chosen the genes appeared more than 3 times. There were 9 related genes: IL-1β, IL- 6, TLR4, caspase 1, spleen associated tyrosine kinase, CD40, C5, CXCL5, lipopolysaccharide binding protein, COL1A1. By q-PCR validation, we found that TLR4 (Fig. 4A), C5 (Fig. 4B), CXCL5 (Fig. 4D), COL1A1 (Fig. 4C) and IL-1β (Fig. 4E) were related to paclitaxel and cisplatin resistant and most of them were immune-related.

### Lower SLC7A2 expression is associated with worse prognosis of NSCLC

**Fig. 5:**
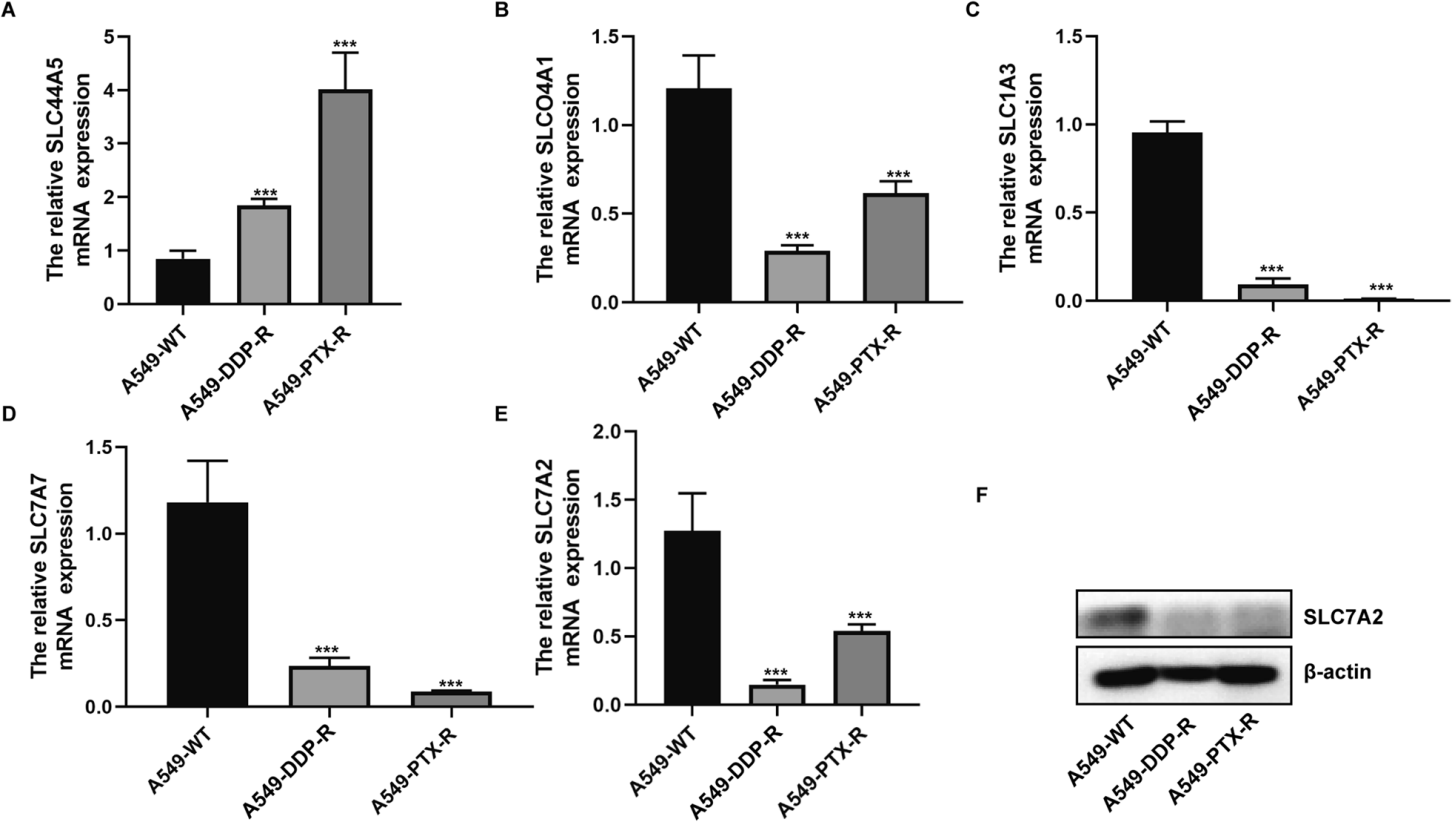
Variable levels of SLC family member expression in drug-resistant A549 cells. The relative levels of SLC44A5 (**A**), SLCO4A1 (**B**), SLC1A3 (**C**), SLC7A7 (**D**), SLC7A2 (**E**) gene transcripts in A549-WT, A549-CIS-R, A549-PTX-R cells were quantified by RT-qPCR. **F** The relative levels of SLC7A2 protein expression in the indicated cell lines were examined by Western blot. Data are representative images or expressed as the mean ± SEM of each group of cells from three separate experiments. ***p < 0.001

It is well known that the transmembrane transport system is vital important in drug transport and resistance, and among them, the solute carrier superfamily (SLC) members are crucial for transporting anti-tumor drugs into cells and lower SLC expression is associated with drug resistance. Accordingly, we analyzed the expression levels of SLC members and found that SLC7A7, SLC7A2, SLCO4A1, SLC1A3 and SLC44A5 were expressed differentially in both resistant cells. RT-qPCR displayed that compared with that in the WT cells, significantly higher levels of SLC44A5, but lower SLC7A7, SLC7A2, SLCO4A1, SLC1A3 mRNA transcripts were detected in both drug-resistant cells (Fig. 5A–E). Given that SLC7A2 is lowly expressed in other types of cancers, such as colorectal cancer and associated with the polarity of macrophage [6, 8]. We chose SLC7A2 for further study in NSCLC. Indeed, Western blot revealed that SLC7A2 protein expression was lower in both drug-resistant cells than that in the WT cells (Fig. 5F).

**Fig. 6:**
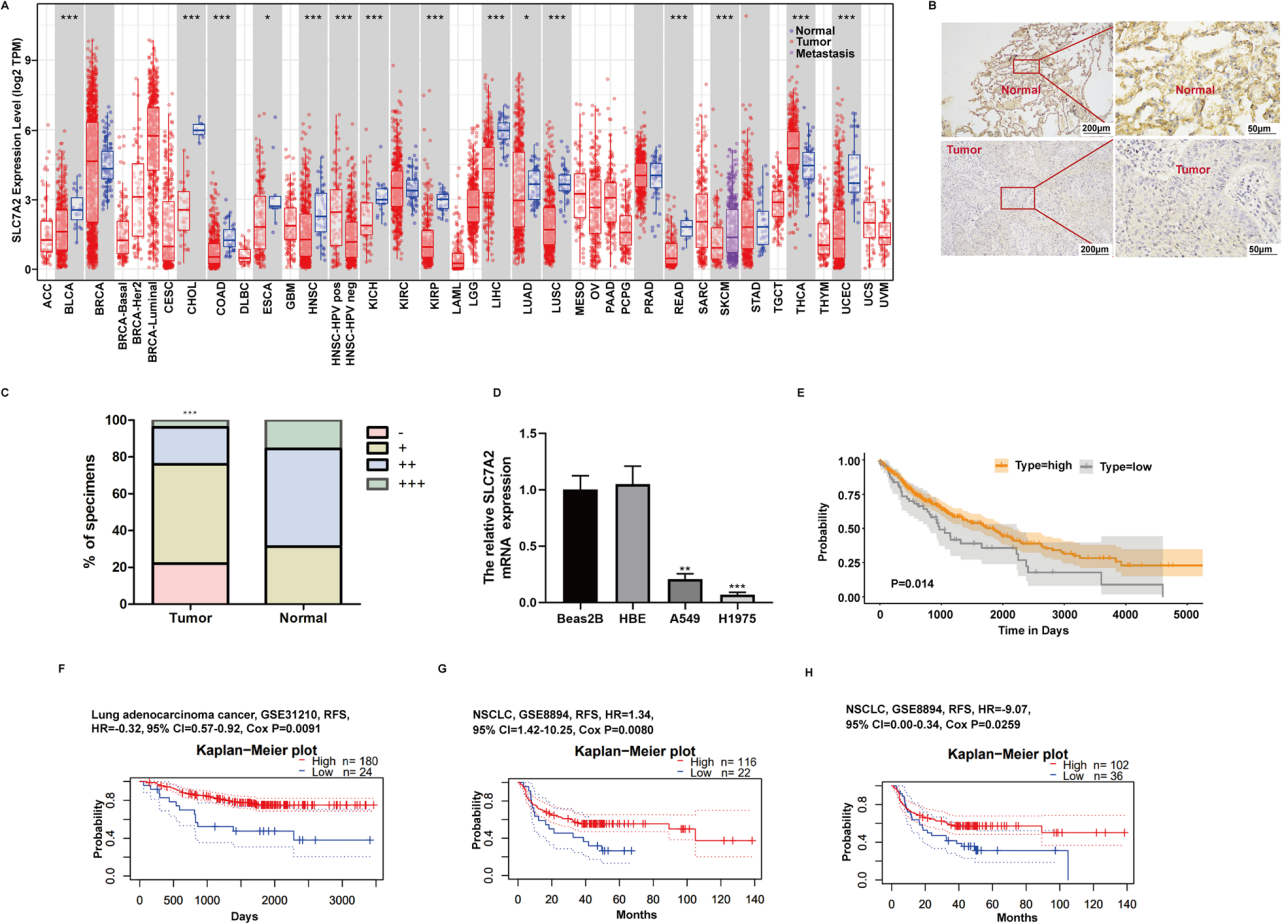
Lower SLC7A2 expression is associated with worse RFS in NSCLC. **A** SLC7A2 mRNA transcripts were down-regulated in various cancers of TCGA in the TIMER database. **B** Tissue specimens were from one patient (magnification, left: × 100 and right: × 400). **C** The percentage of SLC7A2 expression in NSCLC tissue: -, the staining index score < 2; + , 3 < the score < 5; +  + , 6 < the score < 8; +  +  + , 9 < the score < 12. **D** RT-qPCR analysis of the relative levels of SLC7A2 mRNA transcripts in the indicated cell lines. **E** The 427 higher SLC7A2 and 66 lower SLC7A2 expression patients’ prognostic information of Fig. 6A from TCGA database were collected and compared by K–M method of “survival” in R package. Lower SLC7A2 expression group showed poorer prognosis. **F**–**H** Lower SLC7A2 expression was associated with worse RFS of LUAD (GSE31210) or NSCLC (GSE8894)

Analysis of SLC7A2 mRNA transcripts in different types of cancer and adjacent normal tissues in the TIMER database exhibited that the relative levels of SLC7A2 mRNA transcripts in LUAD and LUSC were significantly lower than that in adjacent non-tumor lung tissues (Fig. 6A).

Similar IHC results have been verified by 48 NSCLC patients’ tissues collected from the Shaanxi Provincial People's Hospital in recent 3 years. The SLC7A2 protein expression was obvious lower in tumor tissues than in adjacent non-tumor lung tissues (Fig. 6B, C), and the SLC7A2 expression had no correlation with ages and tumor-node-metastasis stage (Additional file 1: Table S1). Similarly, significantly lower levels of SLC7A2 mRNA transcripts were detected in NSCLC A549 and H1975 cells, relative to that in lung non-tumor Beas2B and HBE cells (Fig. 6D). Next, we investigated whether SLC7A2 was associated with prognosis of NSCLC. The LUSC and LUAD patients’ prognostic information from Fig. 6A (TCGA database) were downloaded by UCSC Xena website (https://xenabrowser.net/). There were 493 patients with survival information among 501 NSCLC patients (427 were higher SLC7A2 expression and 66 were lower expression). By analyzing with K-M method of ‘survminer’ in the R package to generate the survival curves, we found that patients with higher SLC7A2 expression showed better prognosis (Fig. 6E). However, the LUAD prognostic information from TCGA database had no significant difference (Additional file 1: Fig. S2). Moreover, PrognoScan database also showed that lower SLC7A2 expression in lung cancer cohorts (GSE31210, n = 204 and GSE8894, n = 138) was associated with a worse prognosis and the probability of RFS in lower SLC7A2 group was significantly lower than that of those in higher group (HR =  − 0.32, 95% CI = 0.57–0.92, Cox *P* = 0.0091; HR = 1.34, 95% CI = 1.42–10.25, Cox *P* = 0.008; HR =  − 9.07, 95% CI = 0.00–0.34, Cox *P* = 0.0259) (Fig. 6F–H). Therefore, higher SLC7A2 expression may be a protective factor for lung cancer, especially for NSCLC.

### SLC7A2 silencing enhances the proliferation and drug-resistance of NSCLC cells

**Fig. 7:**
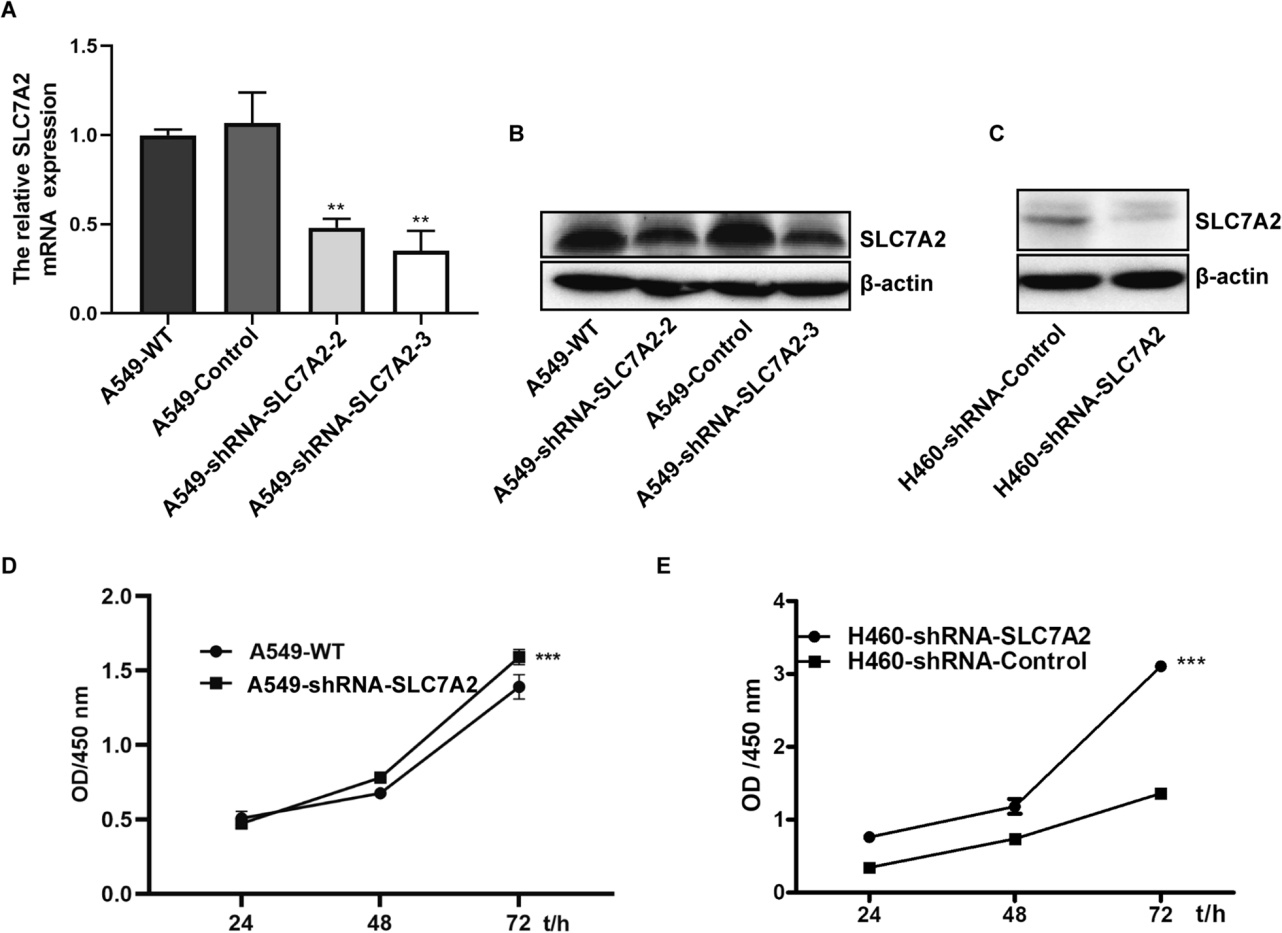
SLC7A2 silencing enhances the proliferation of NSCLC cells. **A**–**C** RT-q-PCR and Western blot analyses of the relative levels of SLC7A2 expression in A549-shRNA-SLC7A2 and H460-shRNA-SLC7A2 cells. **D**, **E** CCK-8 assays indicated that SLC7A2 silencing promoted the proliferation of the indicated cells. Data are representative images or expressed as the mean ± SEM of each group of cells from three separate experiments

**Fig. 8:**
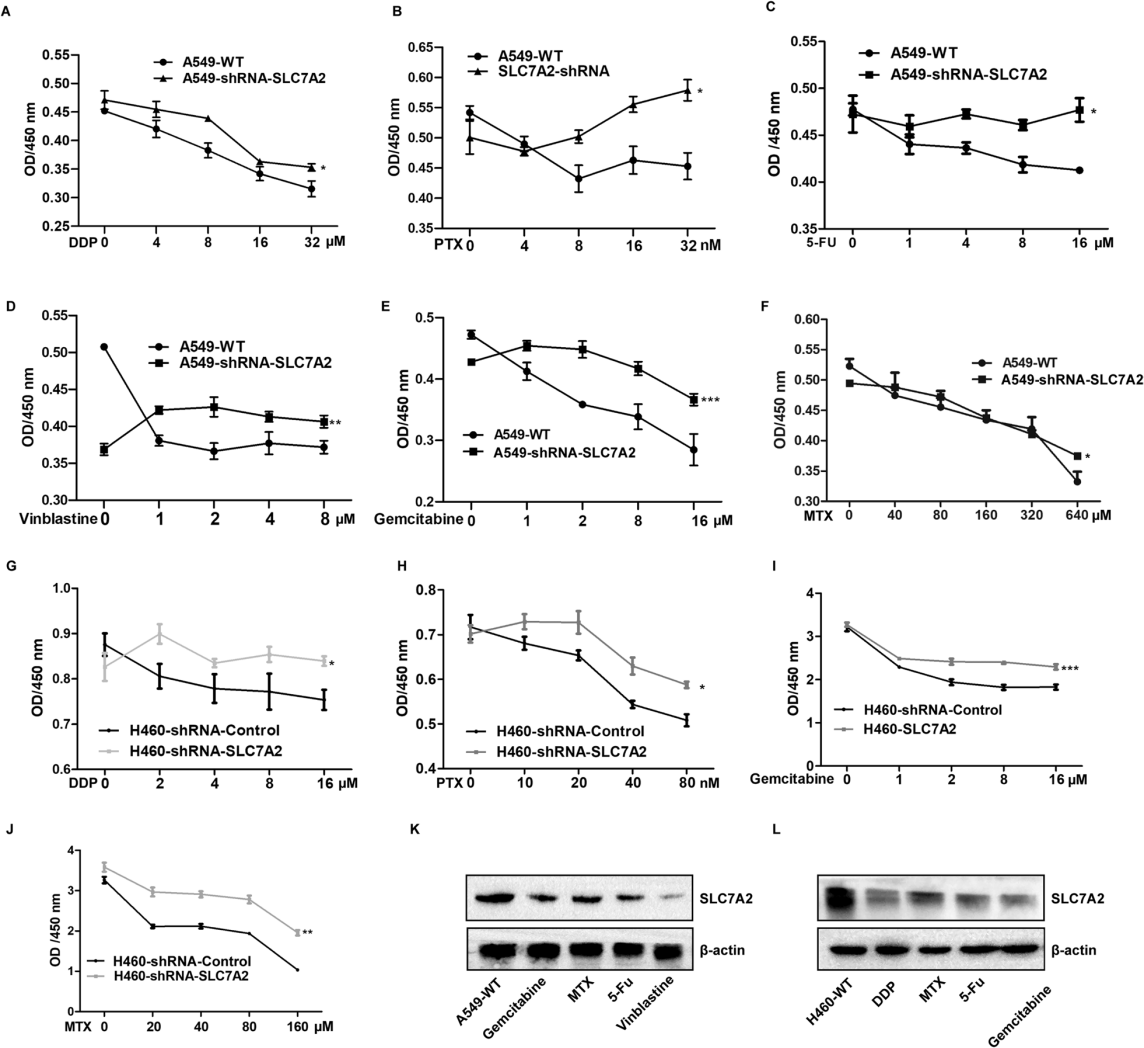
SLC7A2 silencing enhances drug-resistance of NSCLC cells and drug treatment reduces SLC7A2 expression. The dynamic viability and proliferation of the indicated NSCLC cells in the presence of indicated concentrations of the drug were measured by CCK-8 assays. **A**–**F** The dynamic viability of A549-WT and A549-shRNA-SLC7A2. **G**–**J** The dynamic survival of H460-shRNA-Control and H460-shRNA-SLC7A2. **K** A549-WT cells were cultured into 6-cm dishes, and treated with 0.1 µM vinblastine, 3 µM 5-FU, 1 µM gemcitabine and 5 µM MTX for 15 days, respectively. The relative levels of SLC7A2 expression in the cells were measured by Western blot. **L** H460-WT cells were cultured into 6-cm dishes, and treated with 5 µM DDP, 3 nM PTX, 1 µM gemcitabine and 5 µM MTX for 15 days, respectively. The relative levels of SLC7A2 expression in the cells were measured by Western blot. Data are representative images or expressed as the mean ± SEM of each group of cells from three separate experiments

To explore the potential function of SLC7A2 in the development of lung cancer, we established SLC7A2-silenced A549-shRNA-SLC7A2 and H460-shRNA-SLC7A2 cells using lentivirus-based siRNA technology. Western blot and RT-qPCR exhibited significantly lower SLC7A2 expression in A549-shRNA-SLC7A2 and H460-shRNA-SLC7A2 cells, relative to that in the WT controls (Fig. 7A–C). The CCK-8 assays indicated that SLC7A2 silencing dramatically enhanced the proliferation of both types of cells (Fig. 7D, E). Analysis of drug sensitivity revealed that compared with the WT cells, the SLC7A2 silenced A549-shRNA-SLC7A2 cells were significantly less sensitive to 5-FU, cisplatin, paclitaxel, methotrexate, vinblastine and gemcitabine (Fig. 8A–F). Similarly, the SLC7A2 silenced H460-shRNA-SLC7A2 cells were less sensitive to cisplatin, paclitaxel, methotrexate and gemcitabine (Fig. 8G–J). Furthermore, treatment with individual drugs for 15 days decreased the relative levels of SLC7A2 protein expression in both in A549 and H460 cells (Fig. 8K, L). Together, SLC7A2 silencing promoted the proliferation of NSCLC cells, and enhanced their drug resistance in vitro.

### Activation of AMPK up-regulates SLC7A2 expression in NSCLC cells

**Fig. 9:**
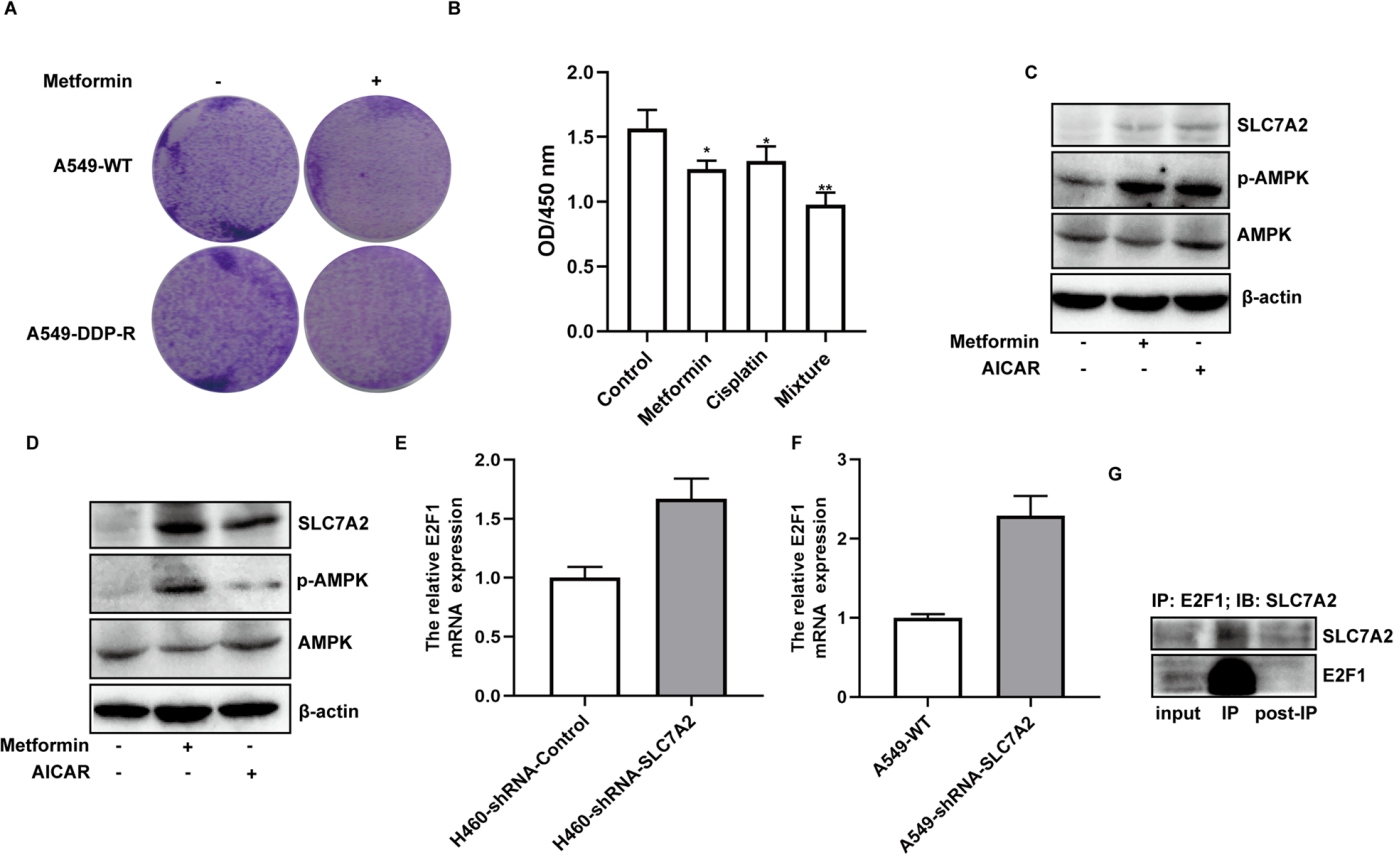
Activation of AMPK up-regulates SLC7A2 expression in NSCLC cells. **A** treatment with metformin reduced the clone formation in both A549-WT and A549-DDP-R cells (magnification × 100). **B** Treatment with metformin and/or cisplatin inhibited the proliferation of H460-WT cells. **C**, **D** Treatment with either metformin or AICAR activated AMPK and up-regulated SLC7A2 expression in A549-WT and H460-WT cells. **E**, **F** The relative E2F1 mRNA expression in H460-shRNA-SLC7A2 and A549-shRNA-SLC7A2 cell lines. **G** the Co-IP assay was performed with H460 cells. And we used E2F1 primary antibody as IP antibody and SLC7A2 primary antibody as IB antibody

Our previous gene array results indicated that metformin could up-regulate SLC7A2 transcription (Additional file 1: Fig. S1A–B), and re-sensitize A549-PTX cells to paclitaxel. We found that treatment with metformin reduced the clone formation in both A549-WT and A549-DDP-R cells (Fig. 9A). In H460 cells, treatment with either metformin or cisplatin inhibited the proliferation of H460 cells and combination of both resulted in an additional effect on inhibiting the proliferation of H460 cells (Fig. 9B). Moreover, treatment with either metformin or AICAR up-regulated SLC7A2 expression in A549 and H460 cells (Fig. 9C, D). Hence, activation of AMPK up-regulated SLC7A2 expression in NSCLC cells. Moreover, to investigate the mechanism of SLC7A2, we predicted the potential upstream transcription factors by PROMOINIT [17, 18] and identified that E2F1 could be one of the upstream transcription factors. Therefore, we first measured the E2F1 mRNA expression in A549-shRNA-SLC7A2 and H460-shRNA-SLC7A2 cell lines, which showed that E2F1 had higher expression when we knocked down SLC7A2 (Fig. 9E, F). The Co-IP assay also demonstrated that E2F1 and SLC7A2 had some potential interaction (Fig. 9G).

### The correlation analysis between SLC7A2 expression and immune infiltration levels in NSCLC

**Fig. 10:**
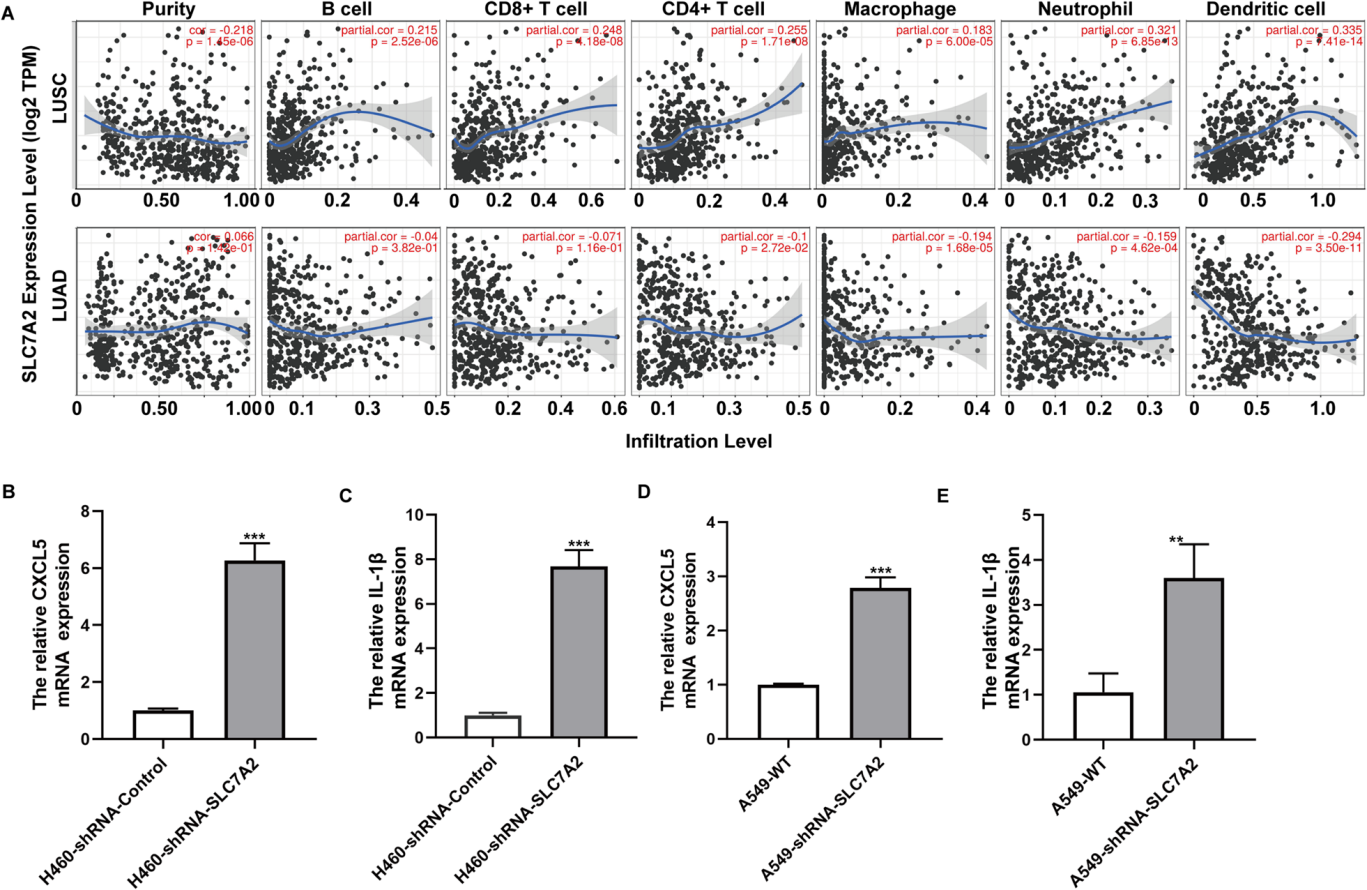
The correlation analysis between SLC7A2 expression and immune infiltration in LUSC and LUAD. **A** The correlation between the levels of SLC7A2 expression and B cell, CD8+ T cell, CD4+ T cell, macrophage, neutrophil and dendritic cell infiltrates in LUSC and LUAD was analyzed. **B**, **C** The relative CXCL5 and IL-1β mRNA expression in H460-shRNA-Control and H460-shRNA-SLC7A2. **D**, **E** The relative CXCL5 and IL-1β mRNA expression in A549-WT and A549-shRNA-SLC7A2

Because there were nearly 200 DEGs related to immune responses in cisplatin- and paclitaxel- resistant NSCLC cells, and tumor infiltrating lymphocytes are crucial for tumor patients’ survival, we tested if SLC7A2 expression could be associated with immune infiltration in NSCLC in the TIMER database. The results indicated that the levels of SLC7A2 transcripts were positively correlated to the levels of macrophage, neutrophil and dendritic cell infiltrates in LUSC and to the tumor purity, B cell, CD8+ T cell, CD4+ T cell, macrophage, neutrophil and dendritic cell infiltrates in LUAD (Fig. 10A). Moreover, the levels of SLC7A2 transcripts were negatively correlated with the levels of CD86, chemokine ligand 2 (CCL2), CSF1R, CD86, IL-10, IRF5, CD163, VSIG4 and MS4A4, but positively with the levels of PTGS2 in LUAD, and with CD86, CCL2, CSF1R, CD86, IL-10, PTGS2, CD163, VSIG4 and MS4A4 in LUSC; CD1C, HLA-DPA1, HLA-DPB1, HLA-DQB1, HLA-DRA and ITGAX in both LUAD and LUSC, with ITGAM and KIR2DL3 in both LUAD and LUSC (Table 1). Therefore, SLC7A2 may promote the infiltration of macrophages, neutrophils and dendritic cells in NSCLC. Moreover, CXCL5 and IL-1β had higher expression in SLC7A2 knock down cell lines (Fig. 10B–E), and both of them were proved to be related to immune infiltration.

**Table 1:** The correlation between SLC7A2 and immune cell markers

Description	Gene	LUAD		LUSC	
		Correlation	*p*	Correlation	*p*
	CCR7	− 0.012	ns	0.289	***
	CEACAM8	− 0.04	ns	0.023	ns
	ITGAM	− 0.27	***	0.341	***
	KIR2DL1	0.131	***	0.119	***
	KIR2DL3	0.073	ns	0.138	***
	KIR2DL4	0.075	ns	0.112	***
	KIR2DS4	0.051	ns	0.205	***
	KIR3DL1	0.04	ns	0.218	***
	KIR3DL2	0.037	ns	0.167	***
	KIR3DL3	0.058	ns	0.082	ns
	CCL2	− 0.22	***	0.325	***
	CD163	− 0.116	***	0.271	***
	CD68	− 0.17	***	0.179	***
	CD86	− 0.247	***	0.275	***
	CSF1R	− 0.244	***	0.318	***
	IL-10	− 0.176	***	0.194	***
	IRF5	− 0.369	***	− 0.047	ns
	MS4A4A	− 0.196	***	0.235	***
	NOS2	0.072	ns	0.064	ns
	PTGS2	0.115	***	0.279	***
	VSIG4	− 0.255	***	0.208	***
	CD1C	− 0.185	***	0.233	***
	HLA-DPA1	− 0.277	***	0.34	***
	HLA-DPB1	− 0.268	***	0.352	***
	HLA-DQB1	− 0.259	***	0.237	***
	HLA-DRA	− 0.311	***	0.327	***
	ITGAX	− 0.15	***	0.319	***
	NRP1	− 0.039	ns	0.25	***

***p < 0.001

## Discussion

Chemotherapy is a big barrier to treatment of NSCLC. Up-regulated expression and function of P-glycoprotein (P-gp) are the most common factors for chemoresistance in tumor cells. Indeed, targeting P-gp by a tyrosine kinase inhibitor pyrazolo [3,4-*d*]pyrimidines can ameliorate chemoresistance [19]. Similarly, inhibition of P-gp expression by BAY-1082439, an inhibitor of PI3K 110α and 110β, also inhibits chemoresistance in tumor cells [20]. Moreover, drug-related nanoparticles can reduce chemoresistance, and increase the efficacy of chemotherapeutic drugs to kill tumor cells [21]. In this study, we generated paclitaxel- or cisplatin-resistant A549 cells and analyzed their transcriptomes by the RNA-Seq assay. We found 223 DEGs in both resistant cells and that most of them were biologically related to the immune system and plasma membrane. On one hand, we explored TLR4, C5, CXCL5, COL1A1 and IL-1β which had relationship with paclitaxel and cisplatin resistant chosen by KEGG related pathways. On the other hand, given that the influx system is very important for transporting anti-tumor drugs into cells, we investigated the expression profiles of the SLC superfamily in NSCLC. The SLC superfamily contains more than 420 members, and functionally transport ions, drugs and other substances. We found that five SLC members had different expression levels in both paclitaxel- and cisplatin-resistant A549 cells. The DEGs were enriched in the immune system in both cisplatin- and paclitaxel-resistant A549 cells. Moreover, lower SLC7A2 has been associated with worse prognosis for some types of cancers and macrophage polarity. Accordingly, we chose SLC7A2 for further researches.

SLC7A2 is a membrane protein and can transport cationic amino acids, like lysine arginine, into cells. Previous studies have demonstrated that lower SLC7A2 expression is associated with worse prognosis of ovarian cancer and hepatocellular carcinoma. In addition, SLC7A2 can down-regulate the expression of CXCL1 to inhibit the infiltration of myeloid-derived suppressor cells and the immune escape of hepatocellular cancer [6]. In contrast, SLC7A2^−/−^ mice display higher levels of proinflammatory chemokines, like CXCL1 and CXCL5, but down-regulated CXCL9 and IL-4 in the tumors, accompanied by increased pro-tumorigenic M2 macrophage activation [8]. In addition, SLC7A2 is a transmembrane transporter, and it may help intracellular delivery of anti-tumor drugs. In this study, we found that SLC7A2 expression was down-regulated in both LUAD and LUSC as well as NSCLC cell line, like A549 and lower SLC7A2 expression was associated with worse RFS in NSCLC patients (GES31210, GES8894 and TCGA database). Hence, SLC7A2 may be a potential biomarker and therapeutic target for NSCLC.

To understand the biological roles of SLC7A2, we generated SLC7A2-silenced A549-shRNA-SLC7A2 and H460-shRNA-SLC7A2 cells and found that SLC7A2 silencing promoted the proliferation of NSCLC cells, suggesting SLC7A2 may be a tumor suppressor in NSCLC. On the other hand, we found that SLC7A2 silencing decreased the sensitivity to 5-FU, cisplatin, paclitaxel, methotrexate, vianblastine and gemcitabine in A549-shRNA-SLC7A2, and to cisplatin, paclitaxel, methotrexate, and gemcitabine in H460-shRNA-SLC7A2 cells, respectively. Interestingly, treatment with each drug for two weeks reduced the expression of SLC7A2 in H460 and A549-WT cells, which may stem from drug-resistance of these cells following long-term exposure. Therefore, SLC7A2 may be crucial for prevention and inhibition of chemoresistance in NSCLC. And the E2F1 could be the important upstream transcription factor in regulating the function of SLC7A2 in drug resistance.

Our previous gene array results indicated that metformin treatment up-regulated SLC7A2 expression. Indeed, we found that treatment with metformin inhibited the clone formation of both wild-type and drug-resistant NSCLC cells and additionally enhanced the cytotoxicity of cisplatin against NSCLC cells. Such data indicated that activation of AMPK by metformin re-sensitized NSCLC cells to inhibition of cisplatin. Consistently, we found that treatment with metformin or another AMPK activator AICAR up-regulated SLC7A2 expression in NSCLC cells. These results indicated that activation of AMPK attenuated chemoresistance in NSCLC partially by up-regulating SLC7A2. AMPK is an energy sensor and acts as a tumor suppressor [22]. It can inhibit mTOR, HIF-1α, JNK, EMT and other signaling pathways [23]. Our previous study has revealed that metformin can increase the sensitivity of A549 cells to paclitaxel by increasing SLCO1B3 expression [16]. The changes in the expression of the SLC members are shown in Additional file 1: Fig. S1. Collectively, the AMPK signaling inhibits the growth of malignant tumors partially by regulating the expression of the SLC superfamily members.

Given that immune infiltrates and their specific types and functions are critical for the prognosis of NSCLC, we analyzed the association of SLC7A2 expression with immune infiltrates in NSCLC. We found that the levels of SLC7A2 mRNA transcripts were correlated with the degrees of macrophage, neutrophil and dendritic cell infiltrates in both LUAD and LUSC. Furthermore, the levels of SLC7A2 mRNA transcripts were positively correlated with the levels of genes for macrophage and dendritic cells in LUAD and LUSC. It is notable that SCL7A2 can shift macrophage activation to pro-tumorigenic M2 during the process of colon tumorigenesis [8]. Consistently, we noticed that the levels of SLC7A2 mRNA transcripts were positively correlated with the levels of CD163, VSIG4 and MS4A4, the M2 macrophage markers in LUSC. These suggest that during the development of NSCLC, lower SLC7A2 expression may change the levels of chemokines to reshape immune landscape in NSCLC to promote its growth and immune escape and modulate its chemoresistance. And the genes of CXCL5 and IL-1β were proved to show higher expression in SLC7A2 knocked down cell lines. In mesenchymal stem cells, IL-1β could enhance IFN-γ receptor expression, then activate STAT5 and p-38-MAPK signaling, at last, increase IL-8 expression and neutrophils recruitment [24]. The activated M1 macrophage cell also secreted IL-1β [25]. CXCL5 could recruit monocytes to form M2-polarized macrophages, weaken the sensitivity of gastric cell line MNK-45 and HGC-27 to 5-FU [26]. With all these results, we could reasonably conclude that SLC7A2 was important in immune infiltration by regulating CXCL5 and IL-1β.

### Limitation

We recognized that our study had limitations. First, although we found that lower SLC7A2 expression was associated with worse prognosis of NSCLC, and its deficiency aggravated chemoresistance, we did not determine whether and how SLC7A2 could modulate the influx transporter of multiple anti-tumor drugs. Second, we only tested the potential function of SLC7A2 in regulating the proliferation, chemosensitivity and clone formation of NSCLC cells. However, we did not analyze the functions of other four SLC members that were differentially expressed in drug-resistant A549 cells. In addition, we found that the levels of SLC7A2 gene transcripts were correlated to the degrees of macrophage, neutrophil and dendritic cell infiltrates and their markers, but on one hand, we only concluded it by bioinformatics and predicted CXCL5 and IL-1β genes were related to immune infiltrate, without animal experiments, which is much more straightforward. Therefore, further investigations on the molecular mechanism underlying the roles of SLC7A2 and other SLC members in regulating the growth, chemoresistance and immune infiltration are warranted in future studies.

## Conclusions

SLC7A2 may act as a tumor suppressor, it was found lower expression in NSCLC tissues and cell lines. Moreover, SLC7A2 could increase drug sensitivity, immune infiltration and survival in NSCLC.

## Supplementary Information

**Additional file 1: Figure S1** **A** The heatmap analyses of the DEGs about SLC superfamily members between A549-WT and A549-WT cells treated with metformin. **B** The relative SLC7A2 mRNA expression in **A**. **Figure S2** The 421 higher SLC7A2 and 92 lower SLC7A2 expressed LUAD patients’ prognostic information of Fig. 6A from TCGA database were collected and compared by the K-M method of ‘survminer’ in the R package, *p* = 0.092. **Table S1** The correlation between SLC7A2 expression and clinical parameters in NSCLC patients

## Data Availability

Not applicable.

## Abbreviations

SLC7A2: Solute carrier family 7 member 2
NSCLC: Non-small-cell lung cancer
RFS: Recurrence-free survival
CCK-8: Cell Counting Kit-8
DEGs: Differentially expressed genes
GO: Gene ontology
KEGG: Kyoto encyclopedia of genes and genomes
MF: Molecular function
CC: Cellular component
BP: Biological process
TIMER: Tumor immune estimation resource
RT-qPCR: Reverse transcription quantitative polymerase chain reaction
