# Supplementary material for: Lower SLC7A2 expression is associated with enhanced multidrug resistance, less immune infiltrates and worse prognosis of NSCLC
Source: Cell Commun Signal. 2023 Jan 13;21:9. doi: 10.1186/s12964-022-01023-x (PMC9838041; doi:10.1186/s12964-022-01023-x)
Supplement: Supplementary file 2 — Additional file 1: Figure S1 A The heatmap analyses of the DEGs about SLC superfamily members between A549-WT and A549-WT cells treated with metformin. B The relative SLC7A2 mRNA expression in A. Figure S2 The 421 higher SLC7A2 and 92 lower SLC7A2 expressed LUAD patients’ prognostic information of Fig. 6A from TCGA database were collected and compared by the K-M method of ‘survminer’ in the R package, p = 0.092. Table S1 The correlation between SLC7A2 expression and clinical parameters in NSCLC patients [file 12964_2022_1023_MOESM2_ESM.docx]

**
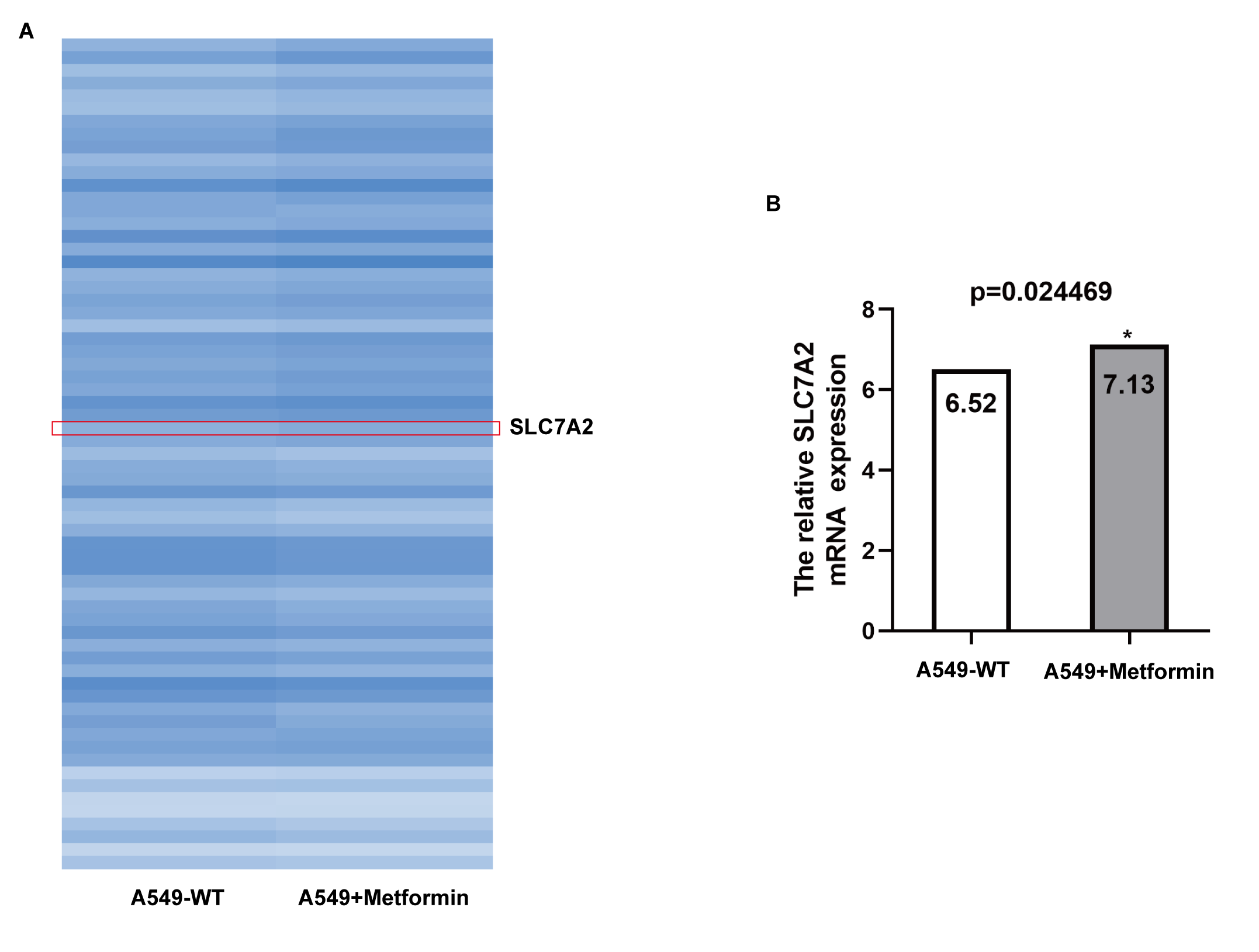
**

**Supplement Figure 1:** A. The heatmap analyses of the DEGs about SLC superfamily members between A549-WT and A549-WT cells treated with metformin. B. The relative SLC7A2 mRNA expression in A.


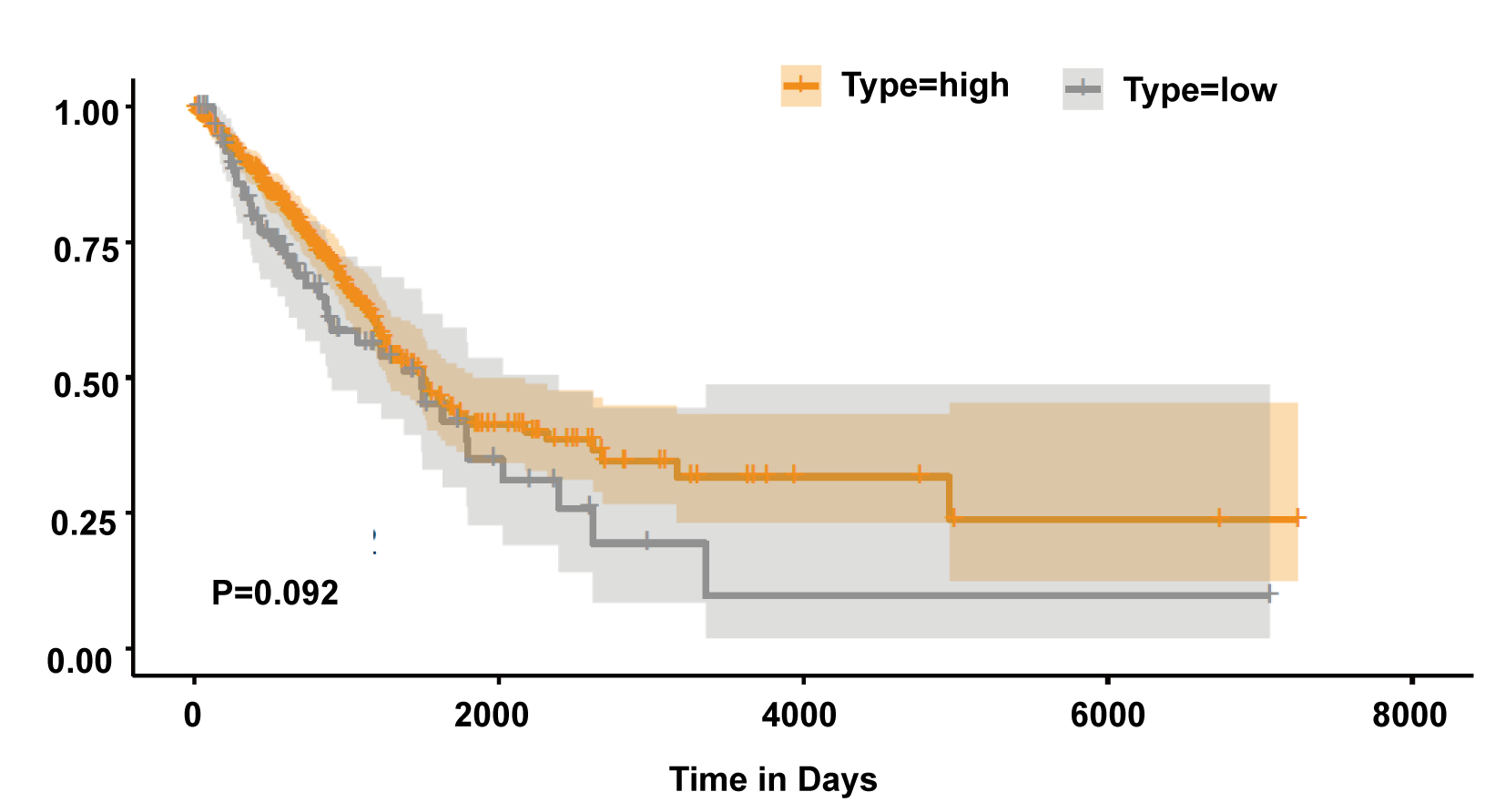


**Supplement Figure 2:** The 421 higher SLC7A2 and 92 lower SLC7A2 expression patients’ prognostic information of Figure 6A from TCGA database were collected and compared by the K-M method of ‘survminer’ in the R package, p=0.092.

**Supplement Table 1**

The correlation between SLC7A2 expression and clinical parameters in NSCLC patients

|  | The expression of SLC7A2 | | | |
| --- | --- | --- | --- | --- |
| Parameter | n | Negative | Positive | p-value |
| Gender |  |  |  | 0.976 |
| Male | 29 | 6 | 23 |  |
| Female | 19 | 4 | 15 |  |
| TNM |  |  |  | 0.552 |
| I and II | 36 | 9 | 27 |  |
| III and IV | 12 | 2 | 10 |  |
